# Determining physiologic variables for changes in 800-m running and 800-m ski ergometer performance

**DOI:** 10.1007/s00421-025-05765-7

**Published:** 2025-04-18

**Authors:** Lars Erik Gjerløw, Arnstein Sunde, Eva Maria Støa, Jan Helgerud, Jan-Michael Johansen, Henrik Hjortland, Øyvind Støren

**Affiliations:** 1 Department of Sports, Physical Education and Outdoor Studies, University of South-Eastern Norway, Bø, Norway; 2 Department of Natural Sciences and Environmental Health, University of South-Eastern Norway, Bø, Norway; 3 Department of Circulation and Medical Imaging, Norwegian University of Science and Technology, Trondheim, Norway; 4 Treningsklinikken, Medical Rehabilitation Clinic, Trondheim, Norway

**Keywords:** Middle-distance performance, Maximal aerobic speed, Maximal sprint speed, Maximal accumulated oxygen deficiency, Anaerobic sprint reserve, Anaerobic capacity

## Abstract

**Purpose:**

This study investigates associations between changes in 800 m time trial performance in running or ski ergometer double poling, and changes in physiologic variables after a seven-week observational period. Forty six athletes ranging from recreational to elite level, participated in either a run (RUN) or a ski ergometer (SKI) observational study.

**Methods:**

The participants performed pre- and post-tests in; 800-m time trial (800TT), 100-m time trial (MSS or MSP), peak oxygen uptake (VO_2peak_), oxygen cost of running (*C*_R_) or double poling (*C*_DP_), time to exhaustion (TTE) at 130% maximal aerobic speed (MAS) or maximal aerobic power (MAP), and maximal accumulated oxygen deficit (MAOD) in SKI. They also performed one repetition maximum (1RM), half-squat (RUN) or 1RM lat pull-down (SKI).

**Results:**

Moderate correlations were found between changes in both MAP and maximal strength and changes in 800TT for SKI (*r = *− 0.51 and *r = *− 0.51, respectively, *p < *0.05). For RUN, MAS and the 0.8 MAS + 0.2 MSS equation correlated (*r = *− 0.71 and *r = *− 0.73, respectively, *p < *0.01) with 800TT. VO_2peak_ was the most important contributor to MAS improvements (RUN) while *C*_DP_ was the most important contributor to MAP improvements (SKI). No correlations were found between changes in TTE at 130% MAS or MAP and, or MAOD, and changes in 800TT, for neither RUN nor SKI. The results from the present study suggest focusing on training to improve maximal oxygen uptake (VO_2max_), work economy and maximal sprint speed to improve performance in middle-distance running and ski sprinting.

**Supplementary Information:**

The online version contains supplementary material available at 10.1007/s00421-025-05765-7.

## Introduction

In competitions lasting approximately 3 min, the athletes will be tested in their ability to sustain an intensity above their maximal aerobic speed (MAS) (Sandford and Stellingwerff 2019). Performance will therefore depend on distributions from both aerobic and anaerobic energy metabolism (Spencer and Gastin 2001; Sandford et al. 2019a). The sprint discipline in cross-country skiing has an average race time of ~ 3 min (Stöggl et al. 2007), giving an ~ 80/20 ratio between aerobic and anaerobic energy contribution respectively (Losnegard et al. 2012). In the 800- and 1500-m middle-distance running, race times vary between approximately 1.5 and 5 min (Sandford and Stellingwerff 2019). Previous studies have examined several physiologic variables likely to determine performance in middle-distance running (Ingham et al. 2008; Sandford et al. 2019a; Bellinger et al. 2021a; Støren et al. 2021; Hallam et al. 2022; Jimenez-Reyes et al. 2022; Watanabe et al. 2024) and sprint cross-country skiing (Stöggl et al 2007; Losnegard et al. 2012; Støren et al. 2023). These variables are i.e., maximal oxygen uptake (VO_2max_), oxygen cost (C) of the given form of movement, maximum sprint speed (MSS), anaerobic capacity measured as maximal accumulated oxygen deficit (MAOD) or time performance in a supra-maximal (related to maximal aerobic capacity) time to exhaustion test (TTE), the capacity to produce, accumulate and exchange and remove lactate, and anaerobic sprint reserve (ASR), meaning the difference between MAS and MSS, different pacing strategies and carnosine content.

Støren et al. (2021, 2023) found MAS and MSS and the equivalent maximal aerobic power (MAP) and maximal sprint power (MSP) to be the main determining factors in 800 m running and 800-m double poling (DP), respectively. This aligns with the results from Bellinger et al. (2021a), but Bellinger et al. (2021a) also found that speed/mechanical characteristics had an impact on the suitability for different 800-m racing strategies and thus results. Neither of these studies found anaerobic endurance capacity measured as either TTE at 130% MAS or MAP or MAOD to impact 800-m performance. This contrasts with Losnegard et al. (2012), who investigated 600-m indoor roller ski performance, as well as with Ramsbottom et al. (1994) and Billat et al. (2009) on 800 m runners, where MAOD correlated negatively with 800-m time. Holsbrekken et al. (2023) showed that competitive skiers had longer time elapsed in a TTE test at the same supra-maximal intensity related to MAP, as well as higher values of accumulated oxygen deficit during intermittent interval bouts, compared to recreational skiers.

The difficulty of measuring anaerobic energetics, as well as both validity and reliability of current measures of anaerobic energy release has previously been discussed (Haugen et al. 2018; Sandford et al. 2021). It is not possible to accurately measure the metabolic cost of work at an intensity above MAS (Saunders et al. 2004), therefore tests assessing anaerobic work will only provide an estimate of anaerobic energy release. For example, MAOD is based on C and the oxygen demand at a given submaximal intensity (Medbø and Tabata 1989; Hill and Vingren 2011).

The concepts of *anaerobic capacity* and *anaerobic endurance capacity* has also been questioned. Both terms are often used to describe the ability to sustain a certain amount of anaerobic energy release over a given period. In addition, anaerobic capacity can also refer to the ability to produce maximal force, maximum speed or maximum jump height, as discussed in Støren et al. (2021) and Støren et al. (2023). Medbø and Tabata (1989) showed that the accumulated oxygen deficiency increased during maximal effort lasting from 30 s to 3 min. Interestingly, when Hill and Vingren (2011) measured MAOD in three all-out tests lasting 3, 5 and 7 min in both cycling and running, MAOD reached approximately the same values in all three tests when MAOD was expressed as absolute numbers. Consequently, MAOD could therefore be seen as a set volume for each individual (Støren et al. 2023; Hill and Vingren 2011). In the study of Støren et al. (2023) MAOD was found to positively correlate with both TTE at 130% MAP as well as with anaerobic power reserve (APR), meaning that a larger APR due to either a relatively lower MAP or a relatively higher MSS would relate to a higher MAOD. Støren et al. (2023) also proposed that the amount of MAOD can be seen as a result of the athlete’s APR, or the equivalent ASR, and therefore determined by the size of the MSP or MSS and the difference between MAP or MSP or MAS and MSS. This was also suggested in Sandford et al. (2019b).

MSS theoretically sets the upper limit for the amount of anaerobic work. Presumably, in two athletes with the same MAS, the athlete with the highest MSS should then have the highest potential for anaerobic work over some time (Sandford et al. 2021; Støren et al. 2023). An indication of this may be the correlation between MAOD (%VO_2peak_) and APR (%MAP) and MAOD (VO_2peak_) and MSP in Støren et al. (2023).

In 2021 and in 2023, Støren et al. presented results showing no relationships between anaerobic capacity measured as either TTE at 130% MAS or MAP or as MAOD, and 800 m time trial (800TT) in running or in DP. This strongly indicated that anaerobic capacity over time, sometimes denoted as anaerobic endurance, had little or no impact on middle-distance time performance in a heterogenous cohort. Of course, since these studies were cross-sectional, little could be shown regarding causality. To our knowledge there has been no other interventional- or observational studies investigating relationships between changes in performance determining factors, i.e., VO_2max_, C, MSS, TTE, and MAOD and changes in middle-distance performance. Therefore, to move one step further in understanding the role of anaerobic capacity in middle-distance disciplines, an observational study on running and DP were conducted. The main purpose of these studies was to investigate possible relationships between changes in 800TT and changes in physiologic variables including TTE and MAOD.

## Methods

### Experimental approach

An observational study with two different samples were conducted to investigate the impact of changes in several physiologic variables on 800TT in running (RUN) and ski ergometer DP (SKI). This study is part of a larger project examining variables associated with performance in middle-distance events in different sports. Consequently, the methods presented here closely align with those detailed in earlier publications from this project (Støren et al. 2021, 2023), however the participants in the present study are new and represent a new data set although this data set like the two previous represent heterogeneity regarding performance. The variables tested were 800TT, VO_2peak_, oxygen cost of running or double poling (*C*_R_ or *C*_DP_), MSS or MSP, maximal strength (1RM), TTE at 130% MAS or MAP and MAOD (only on ski ergometer). The training, and thus observational period lasted for 7 weeks. The two groups of participants performed similar test protocols and observational periods but adjusted to their respective forms of movement.

All tests in the ski ergometer were conducted over two separate days, while all tests in running were conducted over three separate days. After baseline testing, all participants registered their completed training sessions for a 7-week training period before post-testing was conducted. Training was conducted by their own choice and logged in a training registration diary.

### Subjects

A total of 46 (24 women and 22 men) senior athletes from recreational- to elite level volunteered to participate in this study (Table 1). Both RUN and SKI included healthy subjects who either participated in recreational activities i.e., running and strength training, or were long-distance runners at regional to national level, national or elite level cross-country skiers, or elite functional fitness athletes. The heterogeneity in both groups was deliberately chosen to detect different adaptations to different training and performance levels. The study was approved by the institutional research board at the University of Southeastern Norway, the Norwegian Centre for Research Data (NSD, reg 413787 and 183455) and conducted in accordance with the Helsinki declaration. All subjects gave their written consent to participate, after having received information about the study.

**Table 1:** Subject characteristics (*N = *46)

	RUN (*N = *24)	SKI (*N = *22)
Age (years)	28.3 ± 8.1 (28.5)	29.8 ± 10.4 (35.0)
BM (kg)	70.2 ± 13.9 (19.8)	75.4 ± 12.2 (16.2)
Height (cm)	171.8 ± 9.3 (5.4)	174.7 ± 8.1 (4.6)
800TT (s)	185.2 ± 46.4 (25.1)	198.4 ± 30.6 (15.4)
VO_2peak_ (ml∙kg^−1^∙min^−1^)	51.1 ± 11.1 (21.7)	44.2 ± 8.8 (19.9)

Values are mean ± standard deviation, with coefficient of variance in percent in parenthesis

*BM* body mass, *Kg* kilograms, *Cm* centimeters, *800TT* time results in the 800 m, *s* seconds

### Testing procedures

#### Double poling tests

The participants in SKI were tested over 2 different days with at least 1 day in between, and within 2 weeks from the first test, at both pre- and post-testing. All tests except the 1RM pull-down test followed similar procedures as in Støren et al. (2023). On day one the participants were tested for VO_2peak_, *C*_DP_ and TTE. All tests were performed in DP on a ski ergometer (Concept 2 Ski ergometer, Concept2, Vermont, USA). The subjects performed a 10–15-min submaximal workout before the VO_2peak_ test. During the warm-up routine the subjects were instructed to try out and select their preferred flywheel resistance on the ski ergometer. All VO_2_ measurements were performed with the metabolic test system, Jaeger Vyntus CPX (CareFusion, GmbH, Hoechberg, Germany), with a mixing chamber. The subjects began at a starting speed predicted to represent an intensity of approximately 70% of HR_max_. Every 30 s the speed was increased with 0:05 min·500 m^−1^. After 5–7 increases, the subjects were told to progressively increase the speed up to all-out effort and keep this effort until voluntary fatigue. This last stage lasted between 30 and 90 s for all subjects. In addition to voluntary fatigue, heart rate (HR) ≥ 95% of HR_max_, respiratory exchange ratio (RER) ≥ 1.05, as well as a plateau of the VO_2_ curve were used as criteria to evaluate if VO_2peak_ was obtained. The rationale for the specific design of this protocol was previously described and explained in Støren et al. (2023). The mean of the two subsequent highest registered VO_2_-values, each representing 20 s intervals by the mixing chamber, was set as VO_2peak_. All HR measurements were made using Polar s610 HR monitors (Polar Oy, Kempele, Finland). Immediately after the test was completed, a capillary blood sample to measure [La^−^]_b_ was taken. [La^−^]_b_ was measured with a Lactate Scout (SensLab GmbH, Leipzig, ray Inc., Kyoto, Japan).

A one-hour break was given after the VO_2peak_-test was completed. The *C*_DP_-measurement was then carried out as a 5-min work period at a work intensity representing 70–90% of VO_2peak_ obtained from the VO_2peak_ test. The average of five VO_2_ measurements between minutes 3 and 5 (3:30, 3:50, 4:10, 4:30 and 4:50) were used to calculate *C*_DP_ as VO_2_-expenditure expressed in mL⋅kg^−1^⋅w^−1^. Based on the VO_2peak_ and C_DP_ measurements, MAP was calculated as VO_2peak_ ⋅ C_DP_^−1^.

The subjects were tested in TTE 5 min after the *C*_DP_ test. HR and VO_2_ were measured during DP to voluntary exhaustion, at the velocity (in min·500 m^−1^) representing 130% of MAP. Voluntary exhaustion was defined as the point where the subjects made three or more pole strides below the predetermined velocity. [La^−^]_b_ was measured immediately after the test. Both in TTE and 800TT, MAOD was calculated as the mean difference between the mean VO_2_-demand and the mean measured VO_2_. MAOD was expressed both as the product of this difference and time, expressed as mL⋅kg^−1^ and the difference per minute, expressed as mL⋅kg^−1^⋅min^−1^, as well as relative to VO_2peak_ (% VO_2peak_). In the 800TT, VO_2_-demand was calculated as the product of mean race power and the oxygen cost, i.e., W ⋅ *C*_DP_ (Billat et al. 2009). For example, a skier could have a mean 800TT power of 300 W, a *C*_DP_ of 0.250 mL⋅kg^−1^⋅W^−1^, and a race time of 165 s (2.75 min). VO_2_ demand was then 300W ⋅ 0.250 mL⋅kg^−1^⋅W^−1^ = 75 mL⋅kg^−1^⋅min^−1^. If the mean VO_2_ during the 800TT was 58 mL⋅kg^−1^⋅min^−1^, MAOD would be 75–58 = 17 mL⋅kg^−1^⋅min^−1^, or 17 mL⋅kg^−1^⋅min^−1^ ⋅ 2.75 mi*N = *46.75 mL⋅kg^−1^. APR was calculated as the difference of MSP and MAP and is presented in absolute values (W) and as a percent of MAP (%MAP).

The second day of testing consisted of a 100-m time trial (100TT), an 800TT, and a 1RM lat pull-down test. After a 15-min warm up on the ski ergometer, the subjects completed the 100TT as an all-out test. Mean- and peak power, and time performance were measured using the Concept 2 ErgData application (Concept2, Vermont, USA). The MSP was assessed from the peak power measurement during the 100TT. After completing the 100TT, the subjects had an active 10-min break before the 800TT, where they poled at a low intensity of approximately 60% of HR_max_. From the 800TT outlay, the subjects tried to complete the test as fast as possible. HR and VO_2_ was measured continuously, with VO_2_ measurements from the mixing chamber every 10th second. [La^−^]_b_ was measured immediately after the test.

After the 800TT, a 30-min break were given before the 1RM lat pull-down test. Test results from Støren et al. (2008) and Sunde et al. (2010) showed no deterioration in 1RM squat 30 min after VO_2max_ and MAS testing in running and cycling, compared to 1RM without these prior tests. Based on these results it is reasonable to assume that this relationship also applies to the upper body exercises DP in ski ergometer and 1RM lat pull-down. The test was conducted in a pull-down apparatus (Gym2000, Vikersund, Norway). The starting position in the 1RM pull-down test was set with horizontal upper arms and perpendicular elbow joints to determine the position of the bar grip. Each repetition (rep) was approved if the bar passed below the chin in the lower position. The test was built up with several submaximal sets with increasing weight load, progressively approaching 1RM. A 3-min rest was included to recover between sets. The first set was 5 reps at approximately 60% of 1RM, followed by 3 reps at approximately 75% 1RM, 2 reps at approximately 85% 1RM, and 1 rep at approximately 90% 1RM. From there on, sets of 1 repetition at a weight load increased by 2.5–5 kg from the subsequent lift, followed by 3 min of resting, was carried out until 1RM was reached. A similar protocol was previously used in Støren et al. (2008) and Sunde et al. (2010).

#### Running tests

The participants in the RUN-sample were tested on three different days. All three test days were conducted within two weeks of the first test day. Test day 1 consisted of *C*_R_ and VO_2max_ measurements and a 1RM half-squat test, all performed in the laboratory. On test day 2 the participants performed a TTE on an outdoor athletic track. The pace at 130% MAS was calculated based on the *C*_R_ and VO_2max_ measured on test day 1. Test day 3 consisted of two different time trials, the 100TT and 800TT also performed on the outdoor athletic track.

Before the measurement of *C*_R_ and VO_2max_, all participants completed a 10-min warm-up of easy jogging. For both the *C*_R_ and VO_2max_ measurement, a Woodway PPS 55 sport (Waukesha, WI, United States), calibrated for speed and incline, was used. *C*_R_ and VO_2max_ were tested by use of the same metabolic test system as for the DP tests. *C*_R_ was measured at a submaximal steady-state intensity corresponding to 70–90% of VO_2max_. The intensity chosen for the *C* measurements are based on the results from Helgerud et al. (2010), showing the same *C*_R_ at any given running intensity between 70 and 90% of VO_2peak_. Both in RUN and SKI, this intensity averaged 80% of VO_2peak_ with very little variation. The 5-min period was completed at a set velocity, and steady state was ensured by controlling for no additional increase in VO_2_ after 3 of the 5 min. The participants completed two bouts of 5 min with 2 min rest in between, at two different speeds with continuous measurement of VO_2_ and HR. HR was registered with Polar s610 HR monitors (Kempele, Finland). The average of five VO_2_-measurements registered the last minute of each bout were used to calculate *C*_R_ in ml∙kg^−1^∙m^−1^.

The VO_2max_ test was performed within 3 min after the *C*_R_ test. The protocol of the VO_2max_ test was a traditional incremental test to exhaustion at 5% inclination. This protocol was based on the same principles as the VO_2peak_ test conducted in the ski ergometer. The participants began at a starting speed corresponding to an intensity of approximately 80% of HR_max._ The speed was increased every 30 s by 0.5 km∙h^−1^ until voluntary fatigue. The mean of the two highest successive VO_2_-measurements were used to calculate VO_2max_. In addition to voluntary fatigue, HR ≥ 95% of HR_max_, RER ≥ 1.05, as well as a plateau of the VO_2_ curve were used as criteria to evaluate if VO_2peak_ was obtained.

The 1RM half-squat test protocol was similar to the 1RM pull-down test. All participants were given a 30-min break after the VO_2max_-test. The 1RM half-squat test was performed in a Smith-machine (PreCore, Woodinville, WA, United States).

TTE was tested on the second test day. The test was performed on a 400 m outdoor track. For all outdoor tests, also including the 100TT and 800TT, a maximum wind speed of 2.0 m⋅s^−1^ was as standard. In addition to the wind limit, all tests were performed under favorable and similar conditions. To calculate the pace, the MAS expressed in mL⋅kg^−1^⋅m^−1^ was multiplied by 1.3, and later recalculated to km⋅h^−1^. As described in Støren et al. (2021) the 130% MAS was chosen based on the previous work by Blondel et al. (2001), measuring TTE at 120% and 140% of MAS. A cyclist was used to pace the participants during the test. A bike computer was calibrated against the laboratory treadmill to ensure that the correct velocity was being displayed. All participants did a 15–20 min warm-up before the test started. Both the runner and the cyclist used approximately 30 m to accelerate before the starting point. The test ended when the distance between the runner and the cyclist exceeded 2 m. The signal for an ended test were given by one of the timers who were placed around the track. To control that the right average velocity was being upheld during the test, the total distance covered was divided by time spent.

On the third test day, 100TT and 800TT were tested on the outdoor track. The same warm-up routine was used as in test day two. To simulate a competition setting, the participants were placed in heats of two-six persons in both the 100TT and 800TT. The runners started in a standing position, to the sound of a starting pistol. Timers for each runner were placed close to the finish line. After the 100TT, the runners completed an active break of 10 min prior to the 800TT.

In this study, the performance on a 100-m sprint was used as a measure of MSS for the RUN-sample. The rationale for this is previously described in Støren et al. (2021). For the SKI-sample, the peak power in watt on the 100TT was used as a measure of the maximal sprint power (MSP). Based on MAS or MAP and MSS or MSP, ASR or APR was calculated as the difference between these two values. ASR or APR is presented both in absolute values (km⋅h^−1^ or w), in relative values and as a percentage of MAS or MAP.

Test–retest reliability, for the C and VO_2peak_ measurements have in several pilot studies at this lab been shown to be very good, and within 1%. This low variability also lays the basis for reliable MAOD measurements. For the time trials, we cannot rule out, as in any performance test, that the ability to give absolutely all may have some variation from day to day. However, subjective evaluation after each test together with measurements of the VO_2_, [La^−^]_b_ and HR indicated the same effort at the different test days.

### Training registration

Both RUN and SKI completed an average of 7 weeks of training before the post-test. They were not instructed to follow a specific program given by this project. They trained by their own choice. This is therefore an observational study rather than an intervention study. All participants registered their completed exercise sessions in training diaries. They were instructed to use heart rate monitors in every training session. To be able to detect any changes in the training routines, participants also registered their training during the 14 days prior to the pre-test. This is shown in Supplementary tables 4 and 5. The exercise data from the training diaries were categories based on the type of exercise, intensity and the total time elapsed or number of sets and repetitions performed. The different types of exercise were endurance training, strength training, speed training, plyometric training or other training. The endurance training was categorized as either sport specific or general training and divided into two different intensity zones: (1) high-intensity training (> 85% of HR_max_) and (2) low- and moderate intensity training (< 85% of HR_max_). Ski ergometer, cross-country skiing or roller-skiing for SKI, and running for RUN, were defined as specific endurance training, while i.e., cycling, swimming and other were defined as general endurance training. For each participant, intensity zones were calculated using the participant’s individual estimated HR_max,_ based on the heart rate responses during the VO_2peak_-test. The strength training was divided into maximal strength training (> 80% of 1RM or 1–5 RM per set) and other strength training (< 80% of 1RM or ≥ 6 repetitions per set), and also categorized as either upper body strength training or lower body strength training. For the SKI-sample, upper body strength training was further divided into ski-specific exercises, i.e., lat pull-down or chins, or other upper body exercises. The duration of strength training was quantified as total training time by counting 10 s per repetition multiplied with 1.5. A similar method was used in Sandbakk et al. (2016) and Johansen et al. (2020b). The same method for quantifying total training time was used for plyometric training. A total of 10 min per strength training session for warm-up and cool-down were registered as low- and moderate intensity endurance training. For speed training the total training time were quantified as total time sprinting. Warm-up and cool-down in the sprint training sessions were registered in the same way as strength- and plyometric training.

### Statistics

Due to the observational design applied in this study, sample size calculations are challenging. The sample size was calculated based on realistic and possible changes in time performance on the 800TT. An average improvement of 8 s was used as an estimate with a standard deviation of the same as the estimated average improvement, power of 80%, and a significance level of 0.05. Based on these calculations, 16 participants are needed for each of the samples in the present study. A possible dropout rate of approximately 20% was taken into account when the recruitment of participants was conducted. Shapiro–Wilk and QQ-plots were used to test normality for the main variable (800TT), for both samples. Normal distribution was found for the RUN-sample (*p = *0.11), but not the SKI-sample (*p = *0.02). In both samples the participants were divided into two respective groups based on 800TT time performance. The data were found to represent normal distribution for the fastest (*p = *0.07 and *p = *0.13) and slowest (*p = *0.07 and *p = *0.32) in the RUN-sample and the SKI-sample, respectively. In the RUN-sample, as parametric statistics were used, the participants were placed either above (*N = *11) or below (*N = *13) the mean 800TT time. Differences between the slowest and fastest runners were analyzed by an independent sample t test. Paired sample t-tests were used to compare means from before and after observation period. For the SKI-sample, the athletes were divided above (*N = *11) or below (*N = *11) the median 800TT time, as non-parametric statistics were used. Differences between the slowest and fastest skiers were analyzed by a Wilcoxon Rank-Sum test (Mann–Whitney). Wilcoxon signed-rank tests were used to evaluate the comparisons between the results for the physiologic variables before and after the observation period. Although data from the SKI-sample were not found to represent normal distribution for the 800TT-results, the descriptive statistics will be expressed as mean ± standard deviation (SD), and coefficient of variance (%). This way to present the data were chosen because 1) normal distribution were found for both samples when divided into two respective groups (fastest and slowest) and 2) it will make it possible to compare results between the samples in the present study as wells as previous findings presented in Støren et al. (2021, 2023).

Linear regression analyzes were used to evaluate possible relationships between the 800TT, 100TT, TTE or MAOD and the other variables. The same analyzes were performed both for the baseline values and the delta values. All correlations were expressed as the correlation factor r from Pearson’s bivariate tests (correlation tests) and supplemented with the standard error of the estimate (SEE). To investigate possible impact of sex on the correlations, partial correlations controlling for sex were performed. These data were only presented as a few examples, since the partial correlations did not differ significantly for the original correlations. Correlation *r*-values were categorized as, high, 0.8–0.9; moderate, 0.7–0.8; low, 0.6–0.7 (Stöggl et. al, 2007). A *p* value < 0.05 was accepted as statistically significant in all tests. Statistical calculations and analysis were performed using the software program STATA version 18.0 BE (Statistics and Data Science, StataCorp, College Station, Texas, USA).

## Results

Subject characteristics for both samples are presented in Table 1. Performance and physiologic results at baseline are presented in Tables 2 and 3 for RUN and SKI, respectively, both for all participants together and divided based on 800TT performance. There was a significant difference (*p < *0.01) between the slowest (226.1 ± 33.9 s) and fastest (150.7 ± 18.9 s) runners, and the slowest (223.4 ± 23.1 s) and fastest (173.5 ± 8.0 s) skiers.

**Table 2:** Performance and physiologic results at baseline (RUN) (*N = *24)

	All (*N = *24)	800TT > 185 s (*N = *11)	800TT < 185 s (*N = *13)
VO_2peak_			
L⋅min^−1^	3.56 ± 0.91 (25.5)	3.14 ± 0.78 (24.9)	3.91 ± 0.88 (22.5)*
mL⋅kg^−1^⋅min^−1^	51.1 ± 11.1 (21.7)	41.6 ± 4.3 (10.4)	59.1 ± 8.1 (13.8)**
HR (BPM)	191 ± 11 (5.9)	194 ± 8 (4.1)	189 ± 14 (7.2)
RER (VCO_2_ / VO_2_)	1.14 ± 0.05 (4.7)	1.14 ± 0.06 (5.4)	1.13 ± 0.05 (4.0)
C_R_			
mL⋅kg^−1^⋅m^−1^	0.205 ± 0.013 (6.1)	0.211 ± 0.012 (5.7)	0.200 ± 0.011 (5.5)*
MAS			
km∙h^−1^	15.1 ± 3.8 (25.0)	11.8 ± 1.2 (10.4)	17.8 ± 2.9 (16.0)**
m∙min^−1^	250.9 ± 62.8 (25.0)	196.9 ± 20.5 (10.4)	296.6 ± 47.6 (16.0)**
MSS			
km∙h^−1^	24.3 ± 3.5 (14.5)	22.5 ± 3.7 (16.5)	25.8 ± 2.7 (10.3)*
m∙min^−1^	404.6 ± 58.9 (14.5)	375.0 ± 62.0 (16.5)	429.6 ± 44.3 (10.3)*
ASR			
km∙h^−1^	9.2 ± 2.6 (28.2)	10.7 ± 2.8 (26.2)	8.0 ± 1.7 (20.9)**
m∙min^−1^	153.7 ± 43.3 (28.2)	178.2 ± 46.7 (26.2)	133.0 ± 27.7 (20.9)**
%MAS (%)	166.3 ± 27.2 (16.4)	189.9 ± 18.8 (9.9)	146.4 ± 13.5 (9.2)**
Equation			
m∙min^−1^	286.6 ± 64.9 (22.6)	232.5 ± 27.5 (11.8)	332.4 ± 49.8 (15.0)**
800 m			
TT (s)	185.2 ± 46.4 (25.1)	226.1 ± 33.9 (15.0)	150.7 ± 18.9 (12.5)**
%MAS (%)	109.7 ± 8.1 (7.4)	109.8 ± 10.4 (9.4)	109.6 ± 6.1 (5.6)
% MSS (%)	67.4 ± 10.3 (15.3)	58.2 ± 7.0 (12.0)	75.2 ± 4.5 (6.0)**
100 m			
TT (s)	15.2 ± 2.4 (15.8)	16.4 ± 2.7 (16.5)	14.1 ± 1.5 (10.6)*
m∙min^−1^	404.6 ± 58.9 (14.5)	375.0 ± 62.0 (16.5)	429.6 ± 44.3 (10.3)*
%MAS (%)	166.3 ± 27.2 (16.4)	189.9 ± 18.8 (9.9)	146.4 ± 13.5 (9.2)**
TTE at 130% MAS			
s	97.9 ± 49.8 (50.9)	139.0 ± 35.6 (25.6)	63.2 ± 29.2 (46.2)**
1RM squat			
kg	116.8 ± 35.7 (30.6)	116.1 ± 41.3 (35.5)	117.5 ± 31.7 (26.9)

Values are mean ± standard deviation, with coefficient of variance in percent in parenthesis. VO_2peak_, peak oxygen consumption. Equation, predicted velocity at 800 m calculated from 0.2MSS + 0.8MAS for the runners using more than 160 s, and 0.3MSS + 0.7MAS for the runners using less than 160 s

*C*_*R*_ oxygen cost of running, *HR* heart rate, *BPM* beats per minute, *RER* respiratory exchange ratio, *MAS* maximal aerobic speed (VO_2peak_ / C_R_), *MSS* maximal sprint speed, *ASR* anaerobic sprint reserve, *TT* time results in the 800 m or the 100 m, *s* seconds, *TTE at 130% MAS* time to exhaustion at 130%% of MAS, *1RM* one repetition maximum

*p < 0.05 significantly different from > 185 s

**p < 0.01significantly different from > 185 s

**Table 3:** Performance and physiological results at baseline (SKI) (*N = *22)

	All (*N = *22)	800TT > 192 s (*N = *11)	800TT < 192 s (*N = *11)
VO_2peak_			
L⋅min^−1^	3.31 ± 0.74 (22.3)	2.72 ± 0.43 (15.7)	3.90 ± 0.45 (11.4)**
mL⋅kg^−1^⋅min^−1^	44.2 ± 8.8 (19.9)	39.7 ± 5.2 (13.0)	48.7 ± 9.5 (19.6)*
HR (BPM)	185 ± 10 (5.2)	184 ± 9 (5.1)	186 ± 10 (5.4)
RER (VCO_2_ / VO_2_)	1.19 ± 0.06 (5.4)	1.17 ± 0.06 (5.3)	1.21 ± 0.06 (5.2)
C_DP_			
mL⋅kg^−1^⋅w^−1^	0.330 ± 0.124 (37.5)	0.429 ± 0.097 (22.7)	0.230 ± 0.030 (13.1)**
MAP			
W	155.0 ± 67.4 (43.5)	97.9 ± 30.5 (31.1)	212.2 ± 37.7 (17.8)**
MSP			
W	407.1 ± 155.6 (38.2)	276.4 ± 76.1 (27.5)	537.9 ± 86.1 (16.0)**
APR			
W	252.1 ± 103.6 (41,1)	178.5 ± 57.2 (32.1)	325.7 ± 85.8 (26.3)**
0.8MAP + 0.2MSP			
W	205.5 ± 82.2 (40.0)	133.6 ± 37.1 (27.8)	277.3 ± 38.0 (13.7)**
800 m			
TT (s)	198.4 ± 30.6 (15.4)	223.4 ± 23.1 (10.3)	173.5 ± 8.0 (4.6)**
HR (BPM)	182 ± 10 (5.6)	183 ± 9 (5.2)	181 ± 11 (6.2)
Mean Power (w)	206.8 ± 81.2 (39.2)	136.0 ± 39.6 (29.1)	277.5 ± 35.4 (12.8)**
%MAP (%)	136.7 ± 21.6 (15.8)	140.7 ± 24.1 (17.1)	132.7 ± 19.0 (14.3)
% MSP (%)	50.8 ± 7.2 (14.1)	49.4 ± 8.2 (16.5)	52.2 ± 6.1 (11.7)
[La^−^]_b_(mM)	14.6 ± 4.0 (27.4)	14.3 ± 4.6 (32.4)	15.0 ± 3.5 (23.2)
MAOD (mL⋅kg^−1^)	71.0 ± 27.4 (38.7)	80.6 ± 31.0 (38.5)	61.4 ± 20.4 (33.3)
MAOD (mL⋅kg^−1^⋅min^−1^)	21.7 ± 8.0 (36.9)	22.1 ± 8.9 (40.5)	21.3 ± 7.4 (34.6)
MAOD (%VO_2peak_)	168.7 ± 75.7 (44.9)	204.4 ± 78.0 (38.2)	132.9 ± 56.0 (42.2)*
Aerobic metabolism (%)	64.1 ± 9.8 (15.3)	61.8 ± 9.5 (15.4)	66.5 ± 10.0 (15.0)
Anaerobic metabolism (%)	35.9 ± 9.8 (27.4	38.2 ± 9.5 (24.9)	33.5 ± 10.0 (29.8)
100 m			
TT (s)	20.9 ± 3.0 (14.5)	23.4 ± 2.2 (9.6)	18.4 ± 0.8 (4.6)**
Mean Power (w)	340.9 ± 130.1 (38.2)	229.4 ± 66.9 (29.2)	452.5 ± 60.7 (13.4)**
Peak power (w)	407.1 ± 155.6 (38.2)	276.4 ± 76.1 (27.5)	537.9 ± 86.1 (16.0)**
%MAP (%)	274.0 ± 55.7 (20.3)	289.1 ± 54.2 (18.7)	258.8 ± 55.5 (21.4)
TTE at 130% MAP			
s	174.1 ± 107.0 (61.5)	204.4 ± 131.9 (64.5)	143.9 ± 68.2 (47.4)
HR (BPM)	182 ± 9 (5.0)	182 ± 8 (4.6)	183 ± 10 (5.4)
[La^−^]_b_	12.3 ± 3.8 (31.0)	11.5 ± 2.9 (24.8)	13.0 ± 4.6 (35.3)
1RM pull-down			
Kg	83.2 ± 23.0 (27.7)	64.5 ± 12.4 (19.3)	101.8 ± 14.0 (13.7)**

Values are mean ± standard deviation, with coefficient of variance in percent in parenthesis. VO_2peak_, peak oxygen consumption

*C*_*DP*_ oxygen cost of double poling, *HR* heart rate, *BPM* beats per minute, *RER* respiratory exchange ratio, *W* watts, *MAP* maximal aerobic power (VO_2peak_ / C_DP_), *MSP* maximal sprint power, *APR* anaerobic power reserve, *[La*^*−*^*]*_*b*_ blood lactate concentration in millimole⋅L^−1^ (mM), *TT* time results in the 800 m or the 100 m, *s* seconds, *MAOD* mean accumulated oxygen deficit, *TTE at 130% MAP* time to exhaustion at 130% of MAP, *1RM* one repetition maximum

*p < 0.05 different from > 192 s

**p < 0.01 different from > 192 s

Improvements in the 800TT of 5.1% and 3.2% were found for the runners and skiers, respectively. The slowest runners improved by 7.1%, while the fastest runners improved by 1.6% (Table 4). The slowest skiers improved by 4.3%, while the fastest skiers improved by 1.6% (Table 5). Baseline correlations with 800TT, 100TT and TTE for the RUN-sample are presented in Supplementary Table 1. MAS (*r = *− 0.89), MSS (*r = *− 0.75), and the MAS + MSS-equation (*r = *−0.90) all showed negative correlation with 800TT (all at *p < *0.01), meaning a higher respective value would give a better 800-m time. A positive correlation was found between TTE and 800TT (*r = *0.66, *p < *0.01), meaning that a longer TTE would give a longer 800TT. To check if sex would compromise the data, we checked with partial correlations controlling for sex and found the difference between the partial and the actual correlations to be very minor. E.g., MAS and 800TT was *r = *− 0.85, while MSS and 800TT was *r = *− 0.65 and TTE and 800TT was *r = *0.55. The significance levels were the same as in the uncorrected material. The same lack of differences between the actual and the partial correlations corrected for sex were found in the SKI material. MAS was negatively correlated to TTE (− 0.84, *p < *0.01), meaning that the larger MAS, the shorter TTE. For this sample, a higher ASR (expressed as %MAS) was positively correlated to a longer TTE (*r = *0.85, *p < *0.01).

**Table 4:** Changes in physiologic variables before and after observation period (RUN) (*N = *24)

	∆ 800TT < 4,2% (*N = *15)	∆ 800TT > 4,2% (*N = *9)
VO_2peak_		
L⋅min^−1^	0.5 ± 3.7	5.4 ± 6.8*
mL⋅kg^−1^⋅min^−1^	0.1 ± 4.1	6.6 ± 5.9**
HR (BPM)	− 0.2 ± 1.8	− 0.3 ± 2.4
RER (VCO_2_ / VO_2_)	1.4 ± 4.7	− 0.9 ± 5.5
C_R_		
mL⋅kg^−1^⋅m^−1^	− 1.1 ± 2.7	0.8 ± 3.5
MAS		
km∙h^−1^	1.2 ± 3.4	5.8 ± 6.2*
m∙min^−1^	1.2 ± 3.4	5.8 ± 6.2*
MSS		
km∙h^−1^	1.1 ± 2.0	2.7 ± 5.0
m∙min^−1^	1.1 ± 2.0	2.7 ± 5.0
ASR		
km∙h^−1^	1.2 ± 8.2	− 0.9 ± 11.7
m∙min^−1^	1.2 ± 8.2	− 0.9 ± 11.7
%MAS (%)	0.1 ± 3.5	− 2.7 ± 5.8
0.8MAS + 0.2MSS		
m∙min^−1^	1.2 ± 2.5	4.9 ± 5.0*
800 m		
TT (s)	− 1.2 ± 2.1	− 9.1 ± 5.4**
%MAS (%)	0.1 ± 3.6	4.3 ± 4.1*
% MSS (%)	0.1 ± 2.4	7.5 ± 6.8**
100 m		
TT (s)	− 1.1 ± 1.9	− 2.5 ± 4.6
m∙min^−1^	1.1 ± 2.0	2.7 ± 5.0
%MAS (%)	0.1 ± 3.5	− 2.7 ± 5.8
TTE at 130% MAS		
s	1.8 ± 22.7	1.8 ± 13.7
1RM squat		
kg	9.0 ± 13.7	9.0 ± 8.3

Values are mean ± standard deviation. VO2peak, peak oxygen consumption. C_R_, oxygen cost of running. Equation, predicted velocity at 800 m calculated from 0.2MSS + 0.8MAS for the runners using more than 160 s, and 0.3MSS + 0.7MAS for the runners using less than 160 s

*HR* heart rate, *BPM* beats per minute, *RER* respiratory exchange ratio, *MAS* maximal aerobic speed (VO2peak / C_R_), *MSS* maximal sprint speed, *ASR* anaerobic sprint reserve, *TT* time results in the 800 m or the 100 m, *s* seconds, *TTE at 130% MAS* time to exhaustion at 130% of MAS, *1RM* one repetition maximum

*p < 0.05, significantly different from < 4.2%

**p < 0.01, significantly different from < 4.2%

**Table 5:** Changes in physiologic variables before and after observation period (SKI) (*N = *22)

	∆ 800TT < 1.6% (*N = *11)	∆ 800TT > 1.6% (*N = *11)
VO_2peak_		
L⋅min^−1^	− 0.6 ± 3.7	3.4 ± 3.0**
mL⋅kg^−1^⋅min^−1^	− 0.4 ± 4.6	3.9 ± 4.1*
HR (BPM)	− 0.6 ± 1.9	− 0.6 ± 2.2
RER (VCO_2_ / VO_2_)	− 0.9 ± 6.2	− 1.2 ± 4.3
C_DP_		
mL⋅kg^−1^⋅w^−1^	− 4.0 ± 11.6	− 15.1 ± 12.9
MAP		
w	5.0 ± 12.2	25.1 ± 21.7*
MSP		
w	0.5 ± 6.5	13.5 ± 8.1**
APR		
w	− 0.5 ± 11.6	7.2 ± 19.0
0.8MAP + 0.2MSP		
w	2.6 ± 7.6	20.2 ± 13.7**
800 m		
TT (s)	− 0.8 ± 0.5	− 5.1 ± 2.9**
HR (BPM)	0.7 ± 2.4	− 0.1 ± 2.3
Mean Power (w)	2.3 ± 1.4	17.8 ± 11.2**
%MAP (%)	− 1.3 ± 11.6	− 4.0 ± 13.4
% MSP (%)	2.2 ± 6.5	4.5 ± 14.0
[La^−^]_b_(mM)	− 3.1 ± 30.9	− 10.0 ± 22.3
MAOD (mL⋅kg^−1^)	4.3 ± 48.4	1.1 ± 44.2
MAOD (mL⋅kg^−1^⋅min^−1^)	5.2 ± 49.0	6.9 ± 48.4
MAOD (%VO_2peak_)	4.6 ± 47.9	− 2.8 ± 41.8
Aerobic metabolism (%)	2.3 ± 12.8	4.0 ± 18.9
Anaerobic metabolism (%)	4.1 ± 33.8	3.8 ± 37.6
100 m		
TT (s)	− 0.1 ± 1.4	− 3.7 ± 1.7**
Mean power (w)	0.5 ± 4.4	12.1 ± 5.7**
Peak power (w)	0.5 ± 6.5	13.5 ± 8.1**
%MAP (%)	− 3.2 ± 12.3	− 7.2 ± 15.3
TTE at 130% MAP		
S	− 2.7 ± 37.8	− 3.8 ± 54.4
HR (BPM)	− 1.5 ± 3.4	1.2 ± 3.2
[La^−^]_b_	− 4.3 ± 22.3	− 12.0 ± 24.2
1RM pull-down		
kg	3.8 ± 4.6	6.3 ± 3.8

Values are mean ± standard deviation

*VO*_*2peak*_ peak oxygen consumption, *C*_*DP*_ oxygen cost of double poling, *HR* heart rate, *BPM* beats per minute, *RER* respiratory exchange ratio, *W* watts, *MAP* maximal aerobic power (VO_2peak_ / C_DP_), *MSP* maximal sprint power, *APR* anaerobic power reserve, *[La*^*−*^*]*_*b*_ blood lactate concentration in millimole⋅L^−1^ (mM), *TT* time results in the 800 m or the 100 m, *s* seconds, *MAOD* mean accumulated oxygen deficit, *TTE at 130% MAP* time to exhaustion at 130% of MAP, *1RM* one repetition maximum

*p < 0.05, significantly different from preintervention value

**p < 0.01, significantly different from preintervention value

**Fig. 1:**
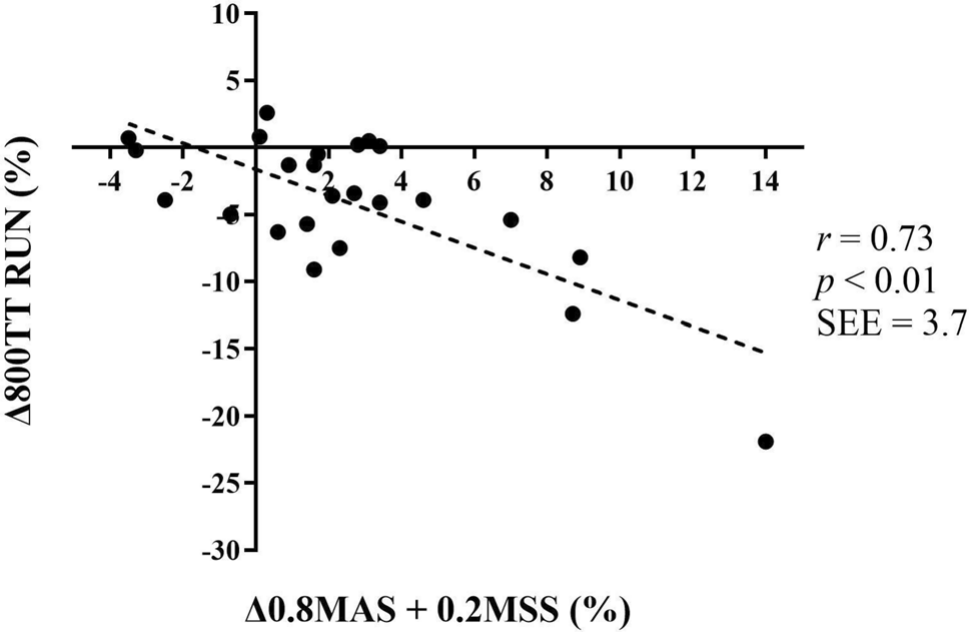
Correlation between changes in 0.8MAS + 0.2MSS and changes in 800TT in RUN. Data are presented as percent changes

Δ MAS and ΔMAS + MSS-equation (*r = *−0.71 and −0.73, respectively, *p < *0.01) (Fig. 1) were found to correlate with Δ800TT (Table 6). In RUN, the improvements in VO_2peak_ accounted for the largest improvement in MAS (*r = *− 0.61, *p < *0.01 with 800TT alone), while C_DP_ accounted for the largest improvements in SKI (*r = *0.47, *p < *0.05 with 800TT alone). The same check for a possible impact of sex as was done in the baseline correlations, was performed for the Δ correlations. Sex had no impact on the correlations here either. E.g., Δ 0.8MAS + 0.2MSS-equation was *r = *− 0.79 (*p < *0.01). The same lack of differences between the actual and the partial Δ correlations corrected for sex were found in the SKI material. No correlation was found between ΔTTE and Δ800TT. Changes in TTE could be related to changes in ASR, both expressed as m∙min^−1^ (*r = *0.56, *p < *0.01) and %MAS (*r = *0.53, *p < *0.01).

**Table 6:** Correlations between changes in different variables (%) and changes in time performances (RUN) (*N = *24)

	800TT (s)	100TT (s)	TTE 130% MAS (s)
VO_2peak_			
L⋅min^−1^	− 0.47 (4.8)*	− 0.09 (3.2)	− 0.29 (19.0)
mL⋅kg^−1^⋅min^−1^	− 0.61 (4.3)**	− 0.08 (3.2)	− 0.35 (18.6)
C_R_			
mL⋅kg^−1^⋅m^−1^	0.00 (5.4)	0.36 (3.0)	− 0.05 (19.9)
MAS			
m∙min^−1^	− 0.71 (3.8)**	− 0.36 (3.0)	− 0.37 (18.4)
MSS			
m∙min^−1^	− 0.40 (4.9)	− 1.0 (0.2)**	0.19 (19.5)
ASR			
m∙min^−1^	0.16 (5.3)	− 0.64 (2.5)**	0.56 (16.5)**
%MAS (%)	0.43 (4.9)*	− 0.33 (3.0)	0.53 (16.8)**
0.8MAS + 0.2MSS			
m∙min^−1^	− 0.73 (3.7)**	− 0.57 (2.6)**	− 0.27 (19.1)
800 m			
TT (s)		0.39 (3.0)	− 0.01 (19.9)
%MAS (%)	0.59 (4.4)**	− 0.14 (3.2)	0.42 (18.0)*
% MSS (%)	− 0.84 (2.9)**	0.17 (3.2)	0.12 (19.7)
100 m			
TT (s)	0.39 (5.0)		− 0.19 (19.5)
m∙min^−1^	− 0.40 (4.9)	− 1.0 (0.2)**	0.19 (19.5)
TTE at 130% MAS			
s	− 0.01 (5.4)	− 0.19 (3.2)	
1RM squat			
kg	− 0.11 (5.3)	− 0.10 (3.3)	− 0.01 (19.3)

Values are the correlation coefficient r, with the standard error of estimate in parenthesis. VO2peak, peak oxygen consumption. . Equation, predicted velocity at 800 m calculated from 0.2MSS + 0.8MAS for the runners using more than 160 s, and 0.3MSS + 0.7MAS for the runners using less than 160 s

*C*_*R*_ oxygen cost of running, *MAS* maximal aerobic speed (VO2peak / C_R_), *MSS* maximal sprint speed, *ASR* anaerobic sprint reserve, *TT* time results in the 800 m or the 100 m, *s* seconds, *TTE at 130% MAS* time to exhaustion at 130% of MAS, *1RM* one repetition maximum

*p < 0.05 significant correlation

** *p < *0.01 significant correlation

Baseline correlations with 800TT, 100TT and TTE for SKI are presented in Supplementary Table 2, and with MAOD in Supplementary Table 3. MAP (*r = *− 0.90), MSP (*r = *− 0.91), 0.8MAP + 0.2MSP (*r = *− 0.93) and 1RM pull-down (*r = *− 0.88) all correlated strongly with 800TT (*p < *0.01). Unlike the results from the RUN-sample, APR showed a moderate negative correlation with 800TT (*r = *− 0.78, *p < *0.01), meaning that a larger APR would relate to a better 800TT performance within the SKI-sample. Strong relationships were found between MAOD, both expressed as mL⋅kg^−1^ (*r = *0.92, *p < *0.01), %VO_2peak_ (*r = *0.90, *p < *0.01) and mL⋅kg^−1^⋅min^−1^ (*r = *0.82, *p < *0.01), and TTE. Neither TTE nor MAOD correlated with 800TT. APR expressed as % MAP correlated moderately with MAOD expressed as % VO_2peak_ (*r = *0.76, *p < *0.01), meaning that the higher APR, the higher MAOD. Similar as in the RUN-sample, strong correlations between TTE and MAOD, both expressed as mL⋅kg^−1^ (*r = *0.85, *p < *0.01), %VO_2peak_ (*r = *0.86, *p < *0.01) and mL⋅kg^−1^⋅min^−1^ (*r = *0.84, *p < *0.01) were found for the SKI-sample.

**Fig. 2:**
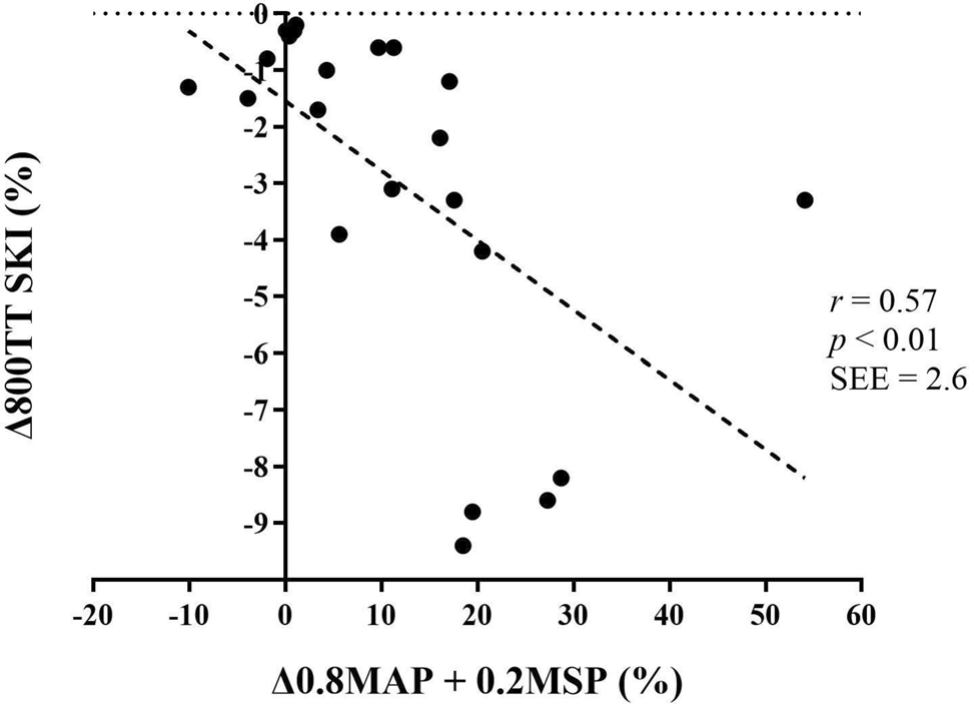
Correlation between changes in 0.8MAP + 0.2MSP and changes in 800TT in SKI. Data are presented as percent changes

Correlations between changes in 800TT, 100TT and TTE and changes in other variables for the SKI-sample are presented in Table 7, and with changes in MAOD in Table 8. Δ800TT correlated with ΔMAP (*r = *− 0.51, *p < *0.05), Δ100TT (s) (*r = *0.77, *p < *0.01) and Δ0.8MAP + 0.2MSP (*r = *− 0.57, *p < *0.01) (Fig. 2). No significant correlations between changes in 800TT and TTE nor MAOD were found.

**Table 7:** Correlations between changes in different variables (%) and changes in time performances (SKI) (*N = *22)

	800TT (s)	100TT (s)	TTE 130% MAP (s)
VO_2peak_			
L⋅min^−1^	− 0.46 (2.8)*	− 0.51 (2.1)*	0.09 (46.7)
mL⋅kg^−1^⋅min^−1^	− 0.34 (2.9)	− 0.41 (2.2)	− 0.01 (46.9)
C_DP_			
mL⋅kg^−1^⋅w^−1^	0.47 (2.7)*	0.42 (2.2)	0.62 (36.9)**
MAP			
w	− 0.51 (2.7)*	− 0.51 (2.1)*	− 0.57 (38.6)**
MSP			
w	− 0.44 (2.8)*	− 0.78 (1.5)**	− 0.26 (45.3)
APR			
w	− 0.02 (3.1)	− 0.37 (2.3)	0.14 (46.4)
0.8MAP + 0.2MSP			
w	− 0.57 (2.6)**	− 0.66 (1.8)**	− 0.54 (39.4)**
800 m			
TT (s)		0.77 (1.5)**	− 0.08 (46.7)
[La^−^]_b_ (mM)	0.10 (3.1)	0.04 (2.4)	− 0.03 (46.9)
MAOD (mL⋅kg^−1^)	− 0.16 (3.1)	0.05 (2.4)	0.85 (24.6)**
MAOD (mL⋅kg^−1^⋅min^−1^)	− 0.24 (3.0)	− 0.10 (2.4)	0.84 (25.1)**
Aerobic metabolism (%)	0.16 (3.1)	− 0.00 (2.4)	− 0.78 (29.5)**
Anaerobic metabolism (%)	− 0.23 (3.0)	− 0.23 (2.4)	0.83 (26.5)**
100 m			
TT (s)	0.77 (2.0)**		0.03 (46.9)
Peak power (w)	− 0.44 (2.8)*	− 0.78 (1.5)**	− 0.26 (45.3)
TTE at 130% MAP			
s	− 0.08 (3.1)	0.03 (2.4)	
[La^−^]_b_ (mM)	0.42 (2.8)	0.42 (2.2)	0.01 (46.9)
1RM pull− down			
kg	− 0.51 (2.7)*	− 0.30 (2.3)	− 0.06 (46.8)

Values are the correlation coefficient r, with the standard error of estimate in parenthesis. VO_2peak_, peak oxygen consumption

*C*_*DP*_ oxygen cost of double poling, *W* watts, *MAP* maximal aerobic power (VO_2peak_ / C_DP_), *MSP* maximal sprint power, *APR* anaerobic power reserve, *[La*^*−*^*]*_*b*_ blood lactate concentration in millimole⋅L^−1^ (mM), *TT* time results in the 800 m or the 100 m, *s* seconds, *MAOD* mean accumulated oxygen deficit, *TTE at 130% MAP* time to exhaustion at 130% of MAP, *1RM* one repetition maximum

*p < 0.05 significant correlation

**p < 0.01 significant correlation

**Table 8:** Correlations between changes in different variables (%) and changes in MAOD (SKI) (*N = *22)

	MAOD (mL⋅kg^−1^)	MAOD (mL⋅kg^−1^⋅min^−1^)	MAOD (%VO_2peak_)
800 m			
TT (s)	0.16 (45.7)	− 0.24 (47.4)	0.12 (44.8)
[La^−^]_b_ (mM)	0.02 (46.3)	0.02 (48.7)	0.02 (45.1)
TTE at 130% MAP			
s	0.85 (24.4)**	0.84 (26.1)**	0.86 (23.4)**
[La^−^]_b_ (mM)	0.07 (46.2)	0.03 (48.7)	0.05 (45.1)
MAP			
w	− 0.53 (39.4)*	− 0.48 (42.9)*	− 0.56 (37.3)**
MSP			
w	− 0.18 (45.6)	− 0.16 (48.1)	− 0.21 (44.1)
APR			
w	0.19 (45.5)	0.17 (48.0)	0.20 (44.2)
%MAP	0.52 (39.6)*	0.47 (43.0)*	0.55 (37.8)**

Values are the correlation coefficient r, with the standard error of estimate in parenthesis. VO_2peak_, peak oxygen consumption

*W* watts, *MAP* maximal aerobic power (VO_2peak_ / C_DP_), *MSP* maximal sprint power, *APR* anaerobic power reserve, *[La*^*−*^*]*_*b*_ blood lactate concentration in millimole⋅L^−1^ (mM), *TT* time results in the 800 m, *S* seconds, *MAOD* mean accumulated oxygen deficit, *TTE at 130% MAP* time to exhaustion at 130% of MAP

*p < 0.05 significant correlation

**p < 0.01 significant correlation

Training characteristics for all participants are presented in Supplementary Table 4 (RUN) and Supplementary Table 5 (SKI).

## Discussion

The main findings in the present study were that changes in 800TT were related to changes in MAS and the MAS + MSS-equation for RUN, while changes in 800TT in DP were shown to significantly correlate with changes in MAP, MSP, and the MAP + MSP-equation as well as changes in 1RM pull-down.

No relationships were found between changes in TTE at 130% MAS or MAP or MAOD, and changes in 800TT in running or DP. This implies that it was the same variables that determined middle-distance performance both at baseline measurements and when correlations between changes in the same variables were conducted after the observational period. Correlations between changes in the different variables measured, and their statistical relationship to each other provides a stronger base for suggesting causality than baseline correlations. The present data is therefore one step further in indicating determining physiologic variables for middle-distance TT performance. As further discussed below, the present results are based on two relatively small cohorts with a large heterogeneity in performance level, and observed over a relative short period of time (7 weeks). Although the present results therefore should be treated with some caution regarding generalization, it is interesting that the correlations between changes in the different variables presented align with baseline correlations in the present study, as well as with baseline correlations in previous studies (Støren et al. 2021, 2023; Støa et al. 2025).

The aerobic endurance, presented as MAS or the equivalent MAP was shown to be a major determinant of 800TT at baseline (*r = *− 0.89, *p < *0.01 and *r = *− 0.90, *p < *0.01, for RUN and SKI, respectively). A significantly (*p < *0.01) higher MAS or MAP was measured for the fastest athletes in both groups at baseline. These findings were in accordance with previous studies investigating sprint skiing performance (Stöggl et al. 2007; Støren et al. 2023) and middle-distance running (Sandford et al. 2019a; Støren et al. 2021; Hallam et al. 2022). Tanji et al. (2018) found a significant negative correlation between C_R_ (*r = *− 0.74), but not VO_2max_, and 800TT in running. The lack of relationship between VO_2max_ and 800 m was later proposed to be a result of homogeneity for this variable in the examined cohort (Støren et al. 2021).

Results in the present study showed that changes in 800TT were related to changes in MAS (*r = *− 0.71, *p < *0.01) and the MAS + MSS-equation (*r = *− 0.73, *p < *0.01). ΔMAS could primarily be explained by the 2.2% average increase in VO_2peak_ (mL⋅kg^−1^⋅min^−1^) from pre- to post-testing. Similar results were found in SKI, as changes in MAP (*r = *− 0.51, *p < *0.05) and 0.8MAP + 0.2MSP (*r = *− 0.57, *p < *0.01) correlated significantly with changes in 800TT. The 0.8MAP + 0.2MSP-equation was based on the expected ~ 80/20 ratio between aerobic and anaerobic energy contribution, respectively, as discussed in Losnegard et al. (2012). The same equation showed significant negative correlation with 800TT in the present study, both at baseline and after the observational period. The 0.8MAP + 0.2MSP-equation was also used as a performance predictor (*r = *− 0.96) in the work of Støren et al. (2023). The finding that MAS or MAP correlated with better 800TT is not surprising. Several previous studies (e.g., Stöggl et al. 2007; Ingham et al. 2008; Sandbakk et al. 2016; Sandford et al. 2019a; Bellinger et al. 2021a, 2021b) have shown a close relationship between MAS or MAP, or velocity at VO_2peak_, and middle-distance performance. A higher MAS or MAP would imply a lower stress of the anaerobic energy systems at a given speed or work. There are two ways to improve MAS, either by improving VO_2max_ or by decreasing the oxygen cost of work (Ingham et al. 2008). Training to improve stroke volume (Helgerud et al. 2007) has been shown to be effective to improve VO_2max_, while training to improve neuro-muscular efficiency (Støren et al. 2008) has been shown effective to lower C.

MSS has been found to be a determining factor of middle-distance performance (Sandford et al. 2019a; Jimenez-Reyes et al. 2022; Støren et al. 2021, 2023). In the present study, significant negative correlations between MSS and MSP and 800TT were found in the baseline results, both for RUN (*r = *− 0.75, *p < *0.01) and SKI (*r = *− 0.91, *p < *0.01), and in the delta results for SKI (*r = *− 0.44, *p < *0.05). The results found in SKI indicate that changes in MSP (*r = *−0.44, *p < *0.05) and 1RM lat pull-down (*r = *− 0.51, *p < *0.05) were of greater importance for the change in 800TT than for the equivalent MSS and 1RM squat for RUN. This may reflect previous findings related to work economy (Støren et al. 2008; Sunde et al. 2010, 2019). With use of lower extremities, Støren et al. (2008) and Sunde et al. (2010) reported lower C after maximal strength training which also improved maximal strength (1RM). However, the improvements in 1RM per se did not correlate with the improvements in C. By use of upper body, Sunde et al. (2019) found a significant relationship with 1RM per se and time performance in DP, and 1RM and C_DP_. The reasons for the difference between the impact of strength in upper- vs. lower body strength may partly be due to that best skiers use the upper body more during training. As for running, the best runners of course often trains more than the slower runners, but the impact of strength may not be as critical since the lower body are a lot more frequently used during daily activities than the upper body.

Anaerobic endurance**,** measured as either TTE or MAOD did not correlate with 800TT for SKI. However, TTE correlated positively with time performance in 800TT (*r = *0.66, *p < *0.01) for RUN. This actually implied that a longer TTE correlated with longer time in 800TT. There may be several possible reasons to why TTE actually correlated with a poorer performance. The main reasons is probably closely related to ASR. As TTE in the present study was performed at 130% MAS. This implies that a high MAS gives a high speed at TTE. In short, a high MAS and a low MSS gives a speed close to MSS, while the opposite is the case when MSS is high and MAS is low. All together, runners or skiers with a high ASR will be better suited to perform a longer time at TTE. On the other hand, runners with a lower MAS may therefore actually perform a longer TTE, which partly is the reason for this finding in the present study. The problem of whether or not ASR is a performance enhancing variable, as previously addressed by Sandford et al. (2019b) and Sandford et al. (2021). To show this point in the present material we performed a partial correlation between baseline TTE and baseline ASR, controlling for MSS. This gave an r-value of 0.86 (*p < *0.01).

The lack of correlations between MAOD and 800TT in the present study was in contrast to results from Losnegard et al. (2012), who found that the fastest skiers in a 600-m indoor roller ski test also produced the larger MAOD. The role of MAOD was previously discussed in Støren et al. (2023) and proposed to be a set volume of anaerobic energy available for each subject. This proposal was supported by the findings in Hill and Vingren (2011), showing approximately the same MAOD in three all-out tests lasting 3, 5 and 7 min in both cycling and running. In Støren et al. (2023) as well as in the SKI-group in the present study, MAOD correlated positively with TTE and APR expressed in % of MAP, suggesting that MAOD could be a product of APR for each athlete. ΔMAOD was also found to correlate positively (*r = *0.85, *p < *0.01) with ΔTTE for the SKI-group in the present study. In the present study, this actually resulted in extreme standard deviations in the changes in MAOD from pre- to post-test. Some of the participants increased MAOD very much when they increased MSP with no increase in MAP. Also, some of the participants decreased MAOD very much after a large improvement in MAP and no change in MSP. In the study by Losnegard et al. (2012), MSP was not presented. It may be that the fastest skiers in Losnegard et al. (2012) had the highest MSP. If so, they may also have had the highest APR, which then would be in line with the present proposal that TTE or MAOD is given by the size of APR.

Holsbrekken et al. (2023) showed that competitive skiers had longer time elapsed in a TTE test at the same supra-maximal intensity related to MAP, as well as higher values of accumulated oxygen deficit during intermittent interval bouts, compared to recreational skiers. Interestingly, accumulated oxygen deficiency (AOD) was measured at a supra-maximal intensity, where work periods of 40 s alternated with recovery periods of 20 s. It was suggested by the authors that the higher AOD measurements could be attributed to the ability to recharge between work phases due to the higher MAP for the fastest skiers. This way of measuring AOD differs from the approach used both in the present study as well as in Losnegard et al. (2012), and the results are therefore not directly comparable. A difference that appears in the protocols used in Støren et al. (2021, 2023) and the present study, compared with the work of Losnegard et al. (2012) and Holsbrekken et al. (2023), is that the latter two studies did not report to have measured MSS. It would have been interesting to see whether a higher MAOD also could be related to a higher sprinting speed, and consequently a possible higher APR, in those studies.

As concluded in del Arco et al. (2022), APR could be a determinant of performance, but only when there are none or minor differences in MAS or MAP or in MSS or MSP. Sandford et al. (2019a) found a negative relationship between ASR and 800TT in a sample with a small standard deviation in MAS. For RUN, ASR expressed as m·min^−1^ did not correlate with 800TT in the present study neither at baseline nor when correlations between changes in these variables were conducted. However, ASR did correlate positively with 800TT when expressed as %MAS (*r = *0.62, *p < *0.01) at baseline, meaning that a higher ASR related to MAS would give a slower time at the 800TT. The opposite was the case in the SKI-group, where a better 800 m performance was related to a larger APR (*r = *− 0.78, *p < *0.01) at baseline. This was also found for the sample studied in Støren et al. (2023). In the present study, the findings in SKI may be explained by the fact that the fastest skiers produced nearly double the watt-values of the slowest skiers at MSP, even though MAP was also found to be significantly larger than for the slowest skiers.

The results in RUN showed that changes in ASR expressed as %MAS correlated positively with changes in 800TT (*r = *0.43, *p < *0.05), meaning that increased ASR after the training period would relate to a decrease in 800TT, for this group. ΔASR was also positively correlated with ΔTTE (*r = *0.56, *p < *0.01), meaning that increased ASR was related to increased time elapsed in the TTE test. APR did not correlate with neither 800TT nor TTE or MAOD for SKI.

A non-significant change in total training volume was found between the period 14 days before pre-tests and the observational period. Although a significant difference was found in plyometric- and maximal strength training volume between the two observed training periods for RUN and SKI, respectively, the absolute values are so low that we assume this increase does not have a major impact on the measured variables at group level. The lack of average change in training volume in the registered categories is likely to be explained by 1) large differences in training volume between the participants and 2) the methodological choice of using an observational period, giving the participants the opportunity to maintain their current training routine throughout the whole period.

### Practical implications, limitations, and future perspectives

The results of the present study give insights into important variables for middle-distance performance in two different forms of movement, running and cross-country skiing. The rationale for including both running and DP in this study, was to be able to see if the results would align in two different types of movement, one with predominantly use of the upper body while the other with predominantly us of the under extremities. While running enabled testing of the 800TT outdoor on a racetrack, DP in a ski ergometer did not have this advantage but enabled measurements of MAOD. In this way the two different types of movements supplemented each other. MAS and MSS for RUN and the equivalent MAP and MSP for SKI showed a strong relationship with performance, while the anaerobic capacity measured as time elapsed at the TTE test and MAOD did not relate to better performances in 800-m running nor ski ergometer DP. It is important to underline that the findings in the present study are found in heterogenous material regarding performance. While heterogeneity is a prerequisite in order to perform correlations, it may deteriorate the specificity toward specific groups of athletes like elite runners or skiers. However, in a recent study (Støa et al. 2025), we echoed the same results in cycling with a homogenous group of junior elite cyclists.

The TTE test in SKI was performed 1 h after the incremental VO_2peak_ test the same day. Although pilot testing prior to the study (TTE with or without an incremental VO2peak test an hour before) in two subjects did not reveal an impact on the TTE, we cannot rule out that the prior incremental test may have compromised the TTE to some extent. Also, in test day 2 800TT was performed 10 min after 100TT. Prior to the Støren et al. (2021, 2023) studies, we performed pilot testing to evaluate a potential effect of the 100TT on the 800TT. The effect was very minor. Approximately half of the pilots performed slightly better on 800TT after the 100TT. The most experienced athletes in the material also reported one or a few sprints to be a normal part of their warm-up routine before their races. However, we cannot rule out that the prior incremental test may have compromised the TTE to some extent.

Both males and females were included in the present study. We did check for possible impact of sex in the correlations by use of partial correlations corrected for sex. The main findings regarding these correlations were approximately the same, both corrected and uncorrected for sex. However, we argue that when comparing time performance with physiologic variables, this can be done independent of sex. When comparing physiologic variables that may differ in results between males and females with performance, results that also differs between males and females, what compares to the difference in performance results is the physiologic differences per se, and not the sex differences. The Sollie and Losnegard (2022) study is an elegant example of this. In that study, distance covered in a 3-min roller-skiing TT was approximately 20% longer in males than in female elite skiers. The sex difference in VO_2peak_ was the same as the difference in TT.

The work presented in the present study can be seen as a continuation of the efforts to understand the complexity of the middle-distance domain. While previous studies have discussed baseline relationships between middle-distance performance in running (Ingham et al. 2008; Sandford et al. 2019a; Støren et al. 2021; Hallam et al. 2022; Jimenez-Reyes et al. 2022) and cross-country skiing (Stöggl et al 2007; Losnegard et al. 2012; Støren et al. 2023), and MAS or MAP, MSS or MSP, and anaerobic capacity, the results of the present study also provide insight into which variables matter when performance changes over a short period of time. Both the baseline and the delta-results of this study can be valuable to consider when planning training programs for athletes in middle-distance running and sprint cross-country skiing. Our results suggest that, to improve performance in middle-distance events, the primary focus of training should be on enhancing MAS or MAP and MSS or MSP. High intensity aerobic interval training (Helgerud et al. 2007; Johansen et al. 2020a, b) and maximal strength training (Østerås et al. 2002; Rønnestad et al. 2008; Støren et al. 2008; Sunde et al. 2010) have been shown to effectively improve either MAS or MAP, or and MSS or MSP in running, skiing and cycling. Training should be less oriented toward improving the capacity to sustain supra-maximal intensity, as this appears to be more influenced by ASR or APR.

The RUN-group did not measure MAOD in the present study. The measurement of MAOD would have been interesting both toward the aim of understanding which variables that determine the 800-m running performance, and also in the comparison between the impact of MAOD in running and ski ergometer DP.

A possible next step to better understand middle-distance performance and the role of anaerobic capacity could be the implementation of a training protocol designed to improve anaerobic endurance performance. It would also be beneficial to repeat the present study in more homogeneous groups of athletes like elite runners or skiers.

## Conclusions

Changes in 800 m performance after a 7-week observational period were related to changes in MAS and the MAS + MSS-equation for RUN, and MAP and 0.8MAP + 0.2MSP for SKI. The results from the present study suggest focusing on training to improve MAS or MAP and maximal sprint speed to improve performance in middle-distance running and ski sprinting.

## Supplementary Information

Below is the link to the electronic supplementary material.

Supplementary file1 (DOCX 23 KB)

## Data Availability

The data sets generated during and/or analyzed during the current study are available from the corresponding author on reasonable request.

## Abbreviations

AOD: Accumulated oxygen deficiency
APR: Anaerobic power reserve
ASR: Anaerobic sprint reserve
BM: Body mass
C: Oxygen cost of work
C_DP_: Oxygen cost of double poling
C_R_: Oxygen cost of running
DP: Double poling
HR: Heart rate
HR_max_: Maximum heart rate
HR_peak_: Peak heart rate
[La^−^]_b_: Blood lactate concentration
MAOD: Maximal accumulated oxygen deficiency
MAP: Maximal aerobic power
MAS: Maximal aerobic speed
MSP: Maximal sprint power
MSS: Maximal sprint speed
RER: Respiratory exchange ratio
RM: Repetition maximum
TT: Time trial
TTE: Time to exhaustion
VO_2max_: Maximal oxygen uptake
VO_2peak_: Peak oxygen uptake
W: Watt
