# Supplementary material for: Determining physiologic variables for changes in 800-m running and 800-m ski ergometer performance
Source: Eur J Appl Physiol. 2025 Apr 18;125(10):2805–21. doi: 10.1007/s00421-025-05765-7 (PMC12479658; doi:10.1007/s00421-025-05765-7)
Supplement: Supplementary file 1 — Supplementary file1 (DOCX 23 KB) [file 421_2025_5765_MOESM1_ESM.docx]

**Supplementary tables**

| **Supplementary table 1. Baseline correlations with time performances (RUN) (N=24)** | | | |
| --- | --- | --- | --- |
|  | **800TT (s)** | **100TT (s)** | **TTE 130% MAS (s)** |
| **VO_2peak_** |  |  |  |
| L⋅min^-1^ | -0.62 (37.1)** | -0.73 (1.7)** | -0.48 (44.7)* |
| mL⋅kg^-1^⋅min^-1^ | -0.89 (21.5)** | -0.72 (1.7)** | -0.80 (30.4)** |
| **C**_R_ |  |  |  |
| mL⋅kg^-1^⋅m^-1^ | 0.50 (41.2)* | 0.46 (2.2)* | 0.55 (42,6)** |
| **MAS** |  |  |  |
| m∙min^-1^ | -0.89 (21.4)** | -0.73 (1.7)** | -0.84 (28.0)** |
| **MSS** |  |  |  |
| m∙min^-1^ | -0.75 (31.2)** | -0.99 (0.4)** | -0.43 (46.0)* |
| **ASR** |  |  |  |
| m∙min^-1^  %MAS (%) | 0.27 (45.7)  0.65 (36.0)** | -0.28 (2.3)  0.17 (2.4) | 0.62 (39.8)**  0.85 (26.5)** |
| **Equation**  m∙min^-1^ | -0.90 (20.4)** | -0.81 (1.4)** | -0.78 (31.9)** |
| **800m** |  |  |  |
| TT (s) |  | 0.79 (1.5)** | 0.66 (38.2)** |
| %MAS (%) | -0.18 (46.7) | -0.01 (2.4) | 0.46 (45.2)* |
| % MSS (%) | -0.79 (29.2)** | -0.26 (2.4) | -0.70 (36.6)** |
| **100m** |  |  |  |
| TT (s) | 0.79 (29.1)** |  | 0.41 (46.5)* |
| m∙min^-1^ | -0.75 (31.2)** | -0.99 (0.4)** | -0.43 (46.0)* |
| **TTE at 130% MAS** |  |  |  |
| s | 0.66 (35.6)** | 0.41 (2.2)* |  |
| **1RM squat**  kg | -0.32 (44.5) | -0.58 (2.0)** | -0.10 (49.3) |
| Values are the correlation coefficient r, with the standard error of estimate in parenthesis. VO2peak, peak oxygen consumption. C_R_, oxygen cost of running. MAS, maximal aerobic speed (VO2peak / C_R_). MSS, maximal anaerobic speed. ASR, anaerobic sprint reserve. Equation, predicted velocity at 800m calculated from 0.2MSS + 0.8MAS for the runners using more than 160 s, and 0.3MSS + 0.7MAS for the runners using less than 160 s. TT, time results in the 800m or the 100m. s, seconds. TTE at 130% MAS, time to exhaustion at 130 per cent of MAS. 1RM, one repetition maximum.  *p<0.05 significant correlation  ** p<0.01 significant correlation | | | |

| **Supplementary table 2. Baseline correlations with time performances (SKI) (N=22)** | | | |
| --- | --- | --- | --- |
|  | **800TT (s)** | **100TT (s)** | **TTE 130% MAP (s)** |
| **VO_2peak_** |  |  |  |
| L⋅min^-1^ | -0.85 (16.5)** | -0.77 (2.0)** | -0.39 (101.0) |
| mL⋅kg^-1^⋅min^-1^ | -0.62 (24.6)** | -0.47 (2.8)* | -0.32 (103.9) |
| **C**_DP_ |  |  |  |
| mL⋅kg^-1^⋅w^-1^ | 0.89 (14.3)** | 0.91 (1.3)** | 0.54 (92,5)* |
| **MAP** |  |  |  |
| W | -0.90 (13.8)** | -0.85 (1.6)** | -0.48 (96.2)* |
| **MSP** |  |  |  |
| W | -0.91 (13.2)** | -0.96 (0.9)** | -0.26 (105.9) |
| **APR** |  |  |  |
| W | -0.78 (19.7)** | -0.89 (1.4)** | -0.08 (109.3) |
| **0.8MAP+0.2MSP**  W | -0.93 (11.4)** | -0.92 (1.2)** | -0.41 (99.9) |
| **800m** |  |  |  |
| TT (s) |  | 0.95 (1.0)** | 0.22 (106.9) |
| [La^-^]_b_ (mM) | -0.17 (30.9) | -0.22 (3.0) | 0.16 (108.3) |
| MAOD (mL⋅kg^-1^) | 0.17 (30.9) | 0.21 (3.0) | 0.92 (42.9)** |
| MAOD (mL⋅kg^-1^⋅min^-1^)  Aerobic metabolism (%)  Anaerobic metabolism (%) | -0.18 (30.8)  -0.08 (31.3)  0.08 (31.3) | -0.13 (3.1)  -0.00 (3.1)  0.00 (3.1) | 0.82 (63.1)**  -0.83 (61.7)**  0.83 (61.7)** |
| **100m** |  |  |  |
| TT (s) | 0.95 (10.1)** |  | 0.23 (106.8) |
| Peak power (w) | -0.91 (13.2)** | -0.96 (0.9)** | -0.26 (105.9) |
| **TTE at 130% MAP** |  |  |  |
| s | 0.22 (30.5) | 0.22 (3.0) |  |
| [La^-^]_b_ (mM)  **1RM pull-down**  kg | 0.25 (30.4)  -0.88 (14.8)** | -0.28 (3.0)  -0.90 (1.3)** | 0.24 (106.4)  -0.33 (103.7) |
| Values are the correlation coefficient r, with the standard error of estimate in parenthesis. VO_2peak_, peak oxygen consumption. C_DP_, oxygen cost of double poling. W, watts. MAP, maximal aerobic power (VO_2peak_ / C_DP_). MSP, maximal sprint power. APR, anaerobic power reserve. [La^-^]_b_, blood lactate concentration in millimole⋅L^-1^ (mM). TT, time results in the 800m or the 100m. s, seconds. MAOD, mean accumulated oxygen deficit. TTE at 130% MAP, time to exhaustion at 130 per cent of MAP. 1RM, one repetition maximum.  *p<0.05 significant correlation  ** p<0.01 significant correlation | | | |

| **Supplementary table 3. Baseline correlations with MAOD (SKI) (N=22)** | | | |
| --- | --- | --- | --- |
|  | **MAOD (mL⋅kg^-1^)** | **MAOD (mL⋅kg^-1^⋅min^-1^)** | **MAOD (%VO_2peak_)** |
| 800m |  |  |  |
| TT (s) | 0.17 (27.7) | -0.18 (8.1) | 0.35 (72.7) |
| [La^-^]_b_ (mM) | 0.13 (27.9) | 0.17 (8.1) | 0.12 (77.1) |
| TTE at 130% MAP |  |  |  |
| s | 0.92 (11.0)** | 0.82 (4.7)** | 0.90 (33.8)** |
| [La^-^]_b_ (mM) | 0.25 (27.2) | 0.36 (7.7) | 0.26 (75.0) |
| MAP |  |  |  |
| w | -0.49 (24.6)* | -0.18 (8.1) | -0.66 (58.4)** |
| MSP |  |  |  |
| w | -0.26 (27.2) | 0.08 (8.2) | -0.35 (72.8) |
| APR |  |  |  |
| w | 0.06 (28.1) | 0.24 (8.0) | -0.10 (77.3) |
| %MAP | 0.58 (23.0)** | 0.50 (7.1)* | 0.76 (50.6)** |
| Values are the correlation coefficient r, with the standard error of estimate in parenthesis. VO_2peak_, peak oxygen consumption. W, watts. MAP, maximal aerobic power (VO_2peak_ / C_DP_). MSP, maximal anaerobic power. APR, anaerobic power reserve. [La^-^]_b_, blood lactate concentration in millimole⋅L^-1^ (mM). TT, time results in the 800m. S, seconds. MAOD, mean accumulated oxygen deficit. TTE at 130% MAP, time to exhaustion at 130 percent of MAP.  *p<0.05 significant correlation  ** p<0.01 significant correlation | | | |

| **Supplementary table 4. Training characteristics during the last two weeks before- and during the observation period (RUN) (N=23)** | | | | | | |
| --- | --- | --- | --- | --- | --- | --- |
|  |  | | |  | | |
|  |  |  | **Before observation period** |  | **During observation period** |  |
| **Duration**  **Training (min**·**week^-1^**) |  |  | 2 weeks |  | 7 weeks |  |
| Total training volume |  |  | 276 ± 200 |  | 269 ± 168 |  |
|  |  |  |  |  |  |  |
|  |  |  |  |  |  |  |
|  |  |  |  |  |  |  |
| **Endurance training (min**·**week^-1^)** |  |  |  |  |  |  |
| Running |  |  |  |  |  |  |
| >85% HR_max_ |  |  | 16 ± 21 |  | 21 ± 20 |  |
| <85% HR_max_ |  |  | 205 ± 178 |  | 200 ± 159 |  |
| Other |  |  |  |  |  |  |
| >85% HR_max_ |  |  | 0 ± 0 |  | 0 ± 2 |  |
| <85% HR_max_ |  |  | 8 ± 31 |  | 13 ± 30 |  |
|  |  |  |  |  |  |  |
| **Strength training (min⋅week^-1^)** |  |  |  |  |  |  |
| Lower body exercises |  |  |  |  |  |  |
| >80% 1RM |  |  | 2 ± 5 |  | 2 ± 3 |  |
| <80% 1RM |  |  | 23 ± 40 |  | 14 ± 21 |  |
| Other exercises |  |  |  |  |  |  |
| >80% 1RM |  |  | 1 ± 1 |  | 1 ± 2 |  |
| <80% 1RM |  |  | 22 ± 38 |  | 18 ± 34 |  |
|  |  |  |  |  |  |  |
| **Speed training (min⋅week^-1^)** |  |  |  |  |  |  |
| Running |  |  | 0 ± 0 |  | 0 ± 0 |  |
|  |  |  |  |  |  |  |
|  |  |  |  |  |  |  |
| **Plyometric training (min⋅week^-1^)** | |  | 0 ± 0 |  | 1 ± 2* |  |
|  |  |  |  |  |  |  |
| **Other training (min·week^-1^)** |  |  | 0 ± 0 |  | 0 ± 0 |  |
| Values are mean ± standard deviation. HRmax, maximal heart rate. Min∙week-1, minutes per week.  * P < 0.05, significantly different from before observation period value. | | | | | | |

| **Supplementary table 5. Training characteristics during the last two weeks before- and during the observation period (SKI) (N=22)** | | | | | | |
| --- | --- | --- | --- | --- | --- | --- |
|  |  | | |  | | |
|  |  |  | **Before observation period** |  | **During observation period** |  |
| **Duration**  **Training (min**·**week^-1^**) |  |  | 2 weeks |  | 7 weeks |  |
| Total training volume |  |  | 335 ± 258 |  | 353 ± 322 |  |
|  |  |  |  |  |  |  |
|  |  |  |  |  |  |  |
|  |  |  |  |  |  |  |
| **Endurance training (min**·**week^-1^)** |  |  |  |  |  |  |
| Ski-specific |  |  |  |  |  |  |
| >85% HR_max_ |  |  | 4 ± 8 |  | 8 ± 14 |  |
| <85% HR_max_ |  |  | 90 ± 184 |  | 102 ± 232 |  |
| Other |  |  |  |  |  |  |
| >85% HR_max_ |  |  | 16 ± 19 |  | 21 ± 15 |  |
| <85% HR_max_ |  |  | 133± 126 |  | 149 ± 125 |  |
|  |  |  |  |  |  |  |
| **Strength training (min⋅week^-1^)** |  |  |  |  |  |  |
| Ski-specific upper body exercises |  |  |  |  |  |  |
| >80% 1RM |  |  | 1 ± 3 |  | 2 ± 3* |  |
| <80% 1RM |  |  | 6 ± 8 |  | 7 ± 10 |  |
| Other upper body exercises |  |  |  |  |  |  |
| >80% 1RM |  |  | 2 ± 5 |  | 5 ± 14 |  |
| <80% 1RM |  |  | 49 ± 58 |  | 38 ± 52 |  |
| Lower body exercises |  |  |  |  |  |  |
| >80% 1RM |  |  | 2 ± 3 |  | 3 ± 7 |  |
| <80% 1RM |  |  | 29 ± 40 |  | 18 ± 21 |  |
|  |  |  |  |  |  |  |
| **Speed training (min⋅week^-1^)** |  |  |  |  |  |  |
| Ski-specific |  |  | 0 ± 0 |  | 0 ± 0 |  |
|  |  |  |  |  |  |  |
|  |  |  |  |  |  |  |
| **Plyometric training (min⋅week^-1^)** | |  | 0 ± 0 |  | 0 ± 1 |  |
|  |  |  |  |  |  |  |
| **Other training (min·week^-1^)** |  |  | 4 ± 10 |  | 1 ± 2 |  |
| Values are mean ± standard deviation. HR_max_, maximal heart rate. Min∙week^-1^, minutes per week.  * P < 0.05, significantly different from preintervention value. | | | | | | |
